# *Anaplasma phagocytophilum* in White-tailed Deer

**DOI:** 10.3201/eid1110.041329

**Published:** 2005-10

**Authors:** Robert F. Massung, Joshua W. Courtney, Shannon L. Hiratzka, Virginia E. Pitzer, Gary Smith, Richard L. Dryden

**Affiliations:** * Centers for Disease Control and Prevention, Atlanta, Georgia, USA; † Washington and Jefferson College, Washington, Pennsylvania, USA; ‡ University of Pennsylvania School of Veterinary Medicine, Philadelphia, Pennsylvania, USA

**Keywords:** Anaplasma phagocytophilum, human granulocytic ehrlichiosis, human anaplasmosis, white-tailed deer, dispatch

## Abstract

We examined the reservoir potential of white-tailed deer for *Anaplasma phagocytophilum*. Results suggest that white-tailed deer harbor a variant strain not associated with human infection, but contrary to published reports, white-tailed deer are not a reservoir for strains that cause human disease. These results will affect surveillance studies of vector and reservoir populations.

*Anaplasma phagocytophilum* is an obligate intracellular bacterium and the etiologic agent of human granulocytic anaplasmosis (formerly known as human granulocytic ehrlichiosis). From 1999 to 2003, a total of 1,686 cases of human anaplasmosis were reported in the United States, and >95% of these cases occurred in northeastern or upper midwestern states. Transmission within and between reservoir populations in these regions occurs by *Ixodes scapularis* ticks (*1**,**2*). Infections occur in humans who have been fed upon by infected nymphal or adult ticks. No evidence shows that *A. phagocytophilum* is transmitted transovarially within the tick population; thus, both infected reservoirs and ticks that can transmit the infection must be available to maintain the agent in nature. Three mammalian species are reservoir competent: the white-footed mouse (*Peromyscus leucopus*), raccoon (*Procyon lotor*), and gray squirrel (*Sciurus carolinensis*), although serologic and molecular evidence has suggested that numerous other small, medium, and large mammals may also be reservoirs (*1**,**3*).

Every examined *A. phagocytophilum* sample from a patient with a confirmed case of human granulocytic anaplasmosis from the northeastern or upper midwestern United States has shown identical 16S rRNA sequences. This sequence, referred to as the *A. phagocytophilum* human anaplasmosis (AP-ha) signature sequence, differs by 2 bp from the sequence of the 16S rRNA gene of a variant strain, AP-Variant 1. Recent studies that compared the prevalence of AP-ha to AP-Variant 1 in tick populations showed the variant to be the predominant strain at 2 of 3 sites and suggest that AP-Variant 1 is common in nature (*4**,**5*).

The white-footed mouse serves as a natural reservoir for AP-ha, and laboratory studies have shown that numerous inbred strains of mice (e.g., Balb/C, C3H, DBA/2) are also highly susceptible to infection (*1**,**6**,**7*). In contrast, AP-Variant 1 does not infect the white-footed mouse, DBA/2, and severely immunocompromised (SCID) mice (*8*). These results suggest that rodents are not a natural reservoir for AP-Variant 1 and that alternative reservoir species exist in nature. Previous reports have identified 3 white-tailed deer (*Odocoileus virginiansus*) from Maryland and 2 white-tailed deer in Wisconsin that harbored an agent with a 16S rRNA gene sequence identical to that of AP-Variant 1 (*4**,**9**,**10*). Several previous studies have also suggested that white-tailed deer are a reservoir for the human agent AP-ha (*10**–**12*). These results led to the current study, which was conducted to investigate the relative potential for white-tailed deer to be a reservoir for AP-ha, AP-Variant 1, or both strains. We examined blood samples from white-tailed deer to determine the strains of *A. phagocytophilum* with which these deer were infected and the ticks feeding on these deer to identify strains to which they were exposed.

## The Study

*I. scapularis* ticks were collected from white-tailed deer during controlled hunts at Ridley Creek State Park in Delaware County, Pennsylvania, in December of 2000, 2001, and 2002. Blood samples were also collected from the deer in 2001 and 2002. An unusually high percentage of ticks collected in December 2000 were positive for *A. phagocytophilum* (68 [49.6%] of 137); identification was based on polymerase chain reaction (PCR) amplification of a 546-bp portion of the 16S rRNA gene, as previously described (Table) (*9*). Further analysis showed a strong correlation between the sex of a tick and the probability of being positive for *A. phagocytophilum*. Most of the positive ticks were females; 62 (84.9%) of the 73 female ticks were positive, compared with 6 (9.4%) of 64 males. DNA sequencing of the PCR products showed that 53 (85.5%) of the 62 PCR-positive females harbored AP-Variant 1, while only 9 (14.5%) of 62 were infected with AP-ha. Blood samples were not collected from white-tailed deer in 2000.

**Table:** Results of polymerase chain reaction and DNA sequencing of Anaplasma phagocytophilum from Ixodes scapularis ticks collected from white-tailed deer

Year	No. ticks*	AP-Variant 1, n (%)	AP-ha, n (%)
2000	73 F	53 (72.6)	9 (12.3)
	64 M	4 (6.3)	2 (3.1)
2001	129 F	37 (28.7)	13 (10.1)
	65 M	10 (15.4)	3 (4.6)
2002	4 F	1 (25)	1 (25)
	2 M	0	1 (50)

*All females were either partially or fully engorged.

In 2001, both ticks and white-tailed deer blood samples were collected. Similar to the year 2000 tick results, a higher percentage of female ticks (50 [38.8%] of 129) than male ticks (13 [20%] of 65) were positive for *A. phagocytophilum*. Likewise, DNA sequencing showed that more infected female ticks were positive for AP-Variant 1 (74%) than for AP-ha (26%). Of the 38 white-tailed deer blood samples collected in 2001, 11 (28.9%) were positive for AP-Variant 1 *A. phagocytophilum*; none of the deer were positive for AP-ha. Most of the AP-Variant 1–positive ticks were obtained from positive white-tailed deer, and AP-Variant 1–positive ticks were collected from 8 of 11 positive deer. Of the 3 positive deer on which AP-Variant 1–positive ticks were not found, 1 deer had only 3 male ticks collected, the second had only 2 males collected, and the third had no ticks. Because adult male ticks do not take a blood meal, we were not surprised that these AP-Variant 1–positive deer had no positive ticks.

The collection of samples in December 2002 resulted in very few ticks (n = 6) because low temperatures inhibited tick activity. PCR amplification and DNA sequencing of *A. phagocytophilum* from female ticks (n = 4) showed that 1 tick was positive for AP-Variant 1 and 1 tick was positive for AP-ha (Table). Only 1 of the 2 males that were collected was positive for *A. phagocytophilum*, which sequencing showed to be AP-ha. Blood samples were collected from 24 white-tailed deer in 2002, and 5 (20.8%) of these were positive for *A. phagocytophilum*. Each of these 5 positive samples was AP-Variant 1.

## Conclusions

The differential between the high percentage of AP-Variant 1–positive female ticks (range 25%–72.6%) and the relatively low number of male ticks positive for the variant (<15.4%), combined with the fact that deer were positive for only the variant, suggest that these partially and fully engorged female ticks were acquiring AP-Variant 1 as they fed on the deer. The presence of AP-ha in both male and female ticks that were feeding on deer showed that these deer were frequently exposed to AP-ha. Despite this demonstrated exposure to AP-ha, infections by AP-ha in white-tailed deer were not found. These results suggest that white-tailed deer are a natural reservoir for the variant, and further, that white-tailed deer are not a reservoir for AP-ha strains. These results also suggest that previous serologic studies that identified white-tailed deer as a reservoir for AP-ha strains were likely the result of the cross-reactivity of the anti-AP-Variant 1 serum from deer with the AP-ha antigens used for detection (*10**–**12*). In fact, in a study in which serologic testing showed that 8% of white-tailed deer in Wisconsin were positive for *A. phagocytophilum*, the only 2 samples that were PCR amplified and sequenced were identical to AP-Variant 1 (*10*). Strong antigenic cross-reactivity of the AP-ha and AP-Variant 1 strains would not be surprising, considering their 16S rRNA genes are >99% identical.

Seroprevalence studies for infectious agents using animal reservoir and host populations and PCR amplifications from vector species are commonly used to assess the disease risk for humans in a particular region, particularly for viral and bacterial zoonotic agents. Our results show that animal seroprevalence studies for *A. phagocytophilum* must be carefully evaluated to determine whether the agent inducing the immune response is truly infectious in humans. Our results further show that while PCR studies of ticks may identify *A. phagocytophilum*, DNA sequencing of the PCR products is necessary to differentiate AP-ha and AP-Variant 1 and therefore to assess the potential for human infections. These issues were not addressed in earlier studies and likely resulted in overestimation of the prevalence of AP-ha in nature and in the implied risk for human anaplasmosis. Therefore, future studies of host or vector populations must be evaluated and interpreted carefully, with the knowledge that non–disease-causing variant strains may influence results.

While our results suggest that white-tailed deer are a reservoir for AP-Variant 1, additional studies that examine the interaction of AP-Variant 1 with white-tailed deer populations in other parts of the United States are needed to determine if they correspond to our results from Pennsylvania. Differences in AP-Variant 1 strain composition or local white-tailed deer or *I. scapularis* tick populations may alter the interaction of the bacterial agent, vector, and reservoir. AP-ha strains cause a transient, relatively mild febrile illness with no overt signs of disease in immunocompetent mouse species, including the natural reservoir, *Peromyscus leucopus* (*13*). Inbred laboratory mice infected with AP-ha may remain infected for up to 55 days (*6*), and previous infections induce an immune response that is only partially protective, since mice may be reinfected (*14*). We have not determined whether AP-Variant 1 produces any disease manifestation in white-tailed deer, although the high number of positive deer in the current study suggests that persistent infections, reinfections from feeding ticks, or both mechanisms may be involved in maintenance of AP-Variant 1 in white-tailed deer populations.
